# Approaches to Improve the Quality of Person Re-Identification for Practical Use

**DOI:** 10.3390/s23177382

**Published:** 2023-08-24

**Authors:** Timur Mamedov, Denis Kuplyakov, Anton Konushin

**Affiliations:** 1 Faculty of Computational Mathematics and Cybernetics, Moscow State University, 119991 Moscow, Russia; denis.kuplyakov@graphics.cs.msu.ru (D.K.); anton.konushin@graphics.cs.msu.ru (A.K.); 2 Video Analysis Technologies LLC, 119634 Moscow, Russia; 3 Faculty of Computer Science, National Research University Higher School of Economics, 109028 Moscow, Russia

**Keywords:** person re-identification, self-supervised pre-training, metric learning, computer vision

## Abstract

The idea of the person re-identification (Re-ID) task is to find the person depicted in the query image among other images obtained from different cameras. Algorithms solving this task have important practical applications, such as illegal action prevention and searching for missing persons through a smart city’s video surveillance. In most of the papers devoted to the problem under consideration, the authors propose complex algorithms to achieve a better quality of person Re-ID. Some of these methods cannot be used in practice due to technical limitations. In this paper, we propose several approaches that can be used in almost all popular modern re-identification algorithms to improve the quality of the problem being solved and do not practically increase the computational complexity of algorithms. In real-world data, bad images can be fed into the input of the Re-ID algorithm; therefore, the new Filter Module is proposed in this paper, designed to pre-filter input data before feeding the data to the main re-identification algorithm. The Filter Module improves the quality of the baseline by 2.6% according to the $Rank_{1}$ metric and 3.4% according to the mAP metric on the Market-1501 dataset. Furthermore, in this paper, a fully automated data collection strategy from surveillance cameras for self-supervised pre-training is proposed in order to increase the generality of neural networks on real-world data. The use of self-supervised pre-training on the data collected using the proposed strategy improves the quality of cross-domain upper-body Re-ID on the DukeMTMC-reID dataset by 1.0% according to the $Rank_{1}$ and mAP metrics.

## 1. Introduction

The task of person re-identification (Re-ID) is to identify the same person in different images obtained from different cameras or at different points in time. That is, a set of images is given, which depict different people in different conditions and from different angles. The task is to determine whether two different images refer to the same person or not. This task belongs to metric learning tasks.

This problem arises in the context of video surveillance and security systems, where it is often necessary to track the movement of people in different zones or places using different video cameras. For example, re-identification can be used at airports, malls, stadiums, or other public places where there are many people and few cameras. The task of person Re-ID is practically important for several reasons:

- **Crime Control.** Re-identification algorithms help in identifying criminals and assist investigators in investigating crimes, including the search for missing persons, abductions, and other serious crimes;
- **Improving the retail customer experience.** In retail, person Re-ID algorithms can help to improve the customer experience by providing personalized offers or services using the analysis of a person’s movement around the store;
- **Analysis of sports events.** Re-identification algorithms are able to automate the processes associated with the analysis of the actions of athletes (for example, the analysis of the movement of football players across the field).

The effectiveness of solving the problem under consideration is of great importance for improving security, ensuring public order and optimizing video surveillance systems. In this regard, research in this area continues, and new techniques and algorithms are constantly being developed for more accurate person Re-ID. Since the algorithms for solving the problem under consideration are used in practice, it is also important to take into account the computational complexity of the re-identification methods during research.

Person Re-ID implies working with real-world data; therefore, due to the variety of situations, when solving this task, we have to face a lot of nuances. Conditionally, there are three main problems that arise when solving this task:

- Lots of object occlusions for re-identification: Due to occlusion, the area for re-identification is reduced, and occlusions can make noise, for example, parts of other objects can get into the image;
- Domain gap problem: The domain in the training data is usually different from the real domain in which the algorithm of person Re-ID will work;
- A small amount of data suitable for training and testing re-identification algorithms: Usually, person Re-ID requires data captured in a multi-camera scenario. Such data are difficult to collect and also expensive to mark up.

One obvious approach to tackle these challenges is by training re-identification algorithms using extensive datasets that have the potential to encompass a wide range of real-world scenarios. However, implementing this approach comes with various aspects that render it either practically infeasible or excessively expensive. Firstly, the datasets must exhibit significant diversity, capturing various situations and visual elements, such as lighting, quality, and angles. Secondly, achieving this diversity requires a substantial amount of data, involving several million images that need manual annotation. This process demands the participation of a large number of markers, resulting in considerable monetary costs. Thirdly, as mentioned earlier, collecting data for person Re-ID is highly problematic due to the unique nature of the task, particularly involving multi-camera scenarios.

Researchers have already made attempts to collect and mark up datasets for the problem under consideration [1,2,3,4]. However, as practice has shown, the data presented was not enough to completely solve the problems mentioned above. Therefore, researchers are studying other approaches that will solve the problems mentioned above.

One of the ways to solve the problem of object occlusions is to feed the algorithm, along with the input image, a binary mask that characterizes the position and shape of the human body that needs to be re-identified [5,6]. This approach contributes to a better perception of the information in the image by the neural network. The mask helps the neural network to focus on a particular person rather than on the background or an overlapping object. There are also solutions where semantic segmentation is used instead of the binary masks [7,8,9]. The essence of semantic segmentation is not in the allocation of the necessary object, but in the classification of the masked objects (for example, outerwear, shoes, etc.). This solution allows the neural network to search for a person more confidently, but this method has its drawbacks. To train the neural network to distinguish objects, it is necessary to manually mark them up in the training data, and this is a very difficult and expensive task.

In some works, the problems described above are proposed to be solved with the help of augmentations. For example, [10,11] uses a special Random Erasing strategy [12] as one of the augmentation approaches, the essence of which is as follows. Random figures are added to images of people in arbitrary places, thus simulating occlusions. This approach makes it possible to increase the amount of data for training and, at the same time, partially solves the problem of frequent occlusions.

There is also another approach to solving the problem with a lack of data—the use more advanced loss functions [13,14,15], which allows training the neural network on a smaller number of images, but at the same time maintaining (or even improving) the results. Loss functions play an important role in training the neural network to focus on a particular person. That is why, when using more advanced functions, less data may be required to train the neural network.

Some researchers suggest increasing training datasets at the expense of synthetic data generated either automatically or semi-automatically. Most often, data generation for training occurs using game engines [16] (for example, Unreal Engine) or directly from the games [17] (for example, Grand Theft Auto V). That is, scenes are generated with a predetermined scenario of the characters’ behavior, and marked-up data are collected in almost automatic mode, which can then be used to train re-identification algorithms. Through the use of software tools, researchers have an almost limitless possibility of generating training samples; with the help of game engines, we can set arbitrary behavior of characters, change their clothes, appearance, environment, etc.

In this paper, we propose our approaches to solving the problems mentioned above that arise in the task of person Re-ID. These approaches were the result of our research aimed at improving the quality of re-identification for practical use. In summary, the contributions of this paper are concluded as follows:

- We will show that larger and advanced neural networks are able to solve the problem of person Re-ID better. However, since the task of re-identification is practically important, it is necessary to find a balance between the quality of the algorithm and its speed.
- We will consider the problem of the algorithm’s stability to image changes and noise. In this paper, we propose using the Jensen–Shannon divergence and AugMix augmentation [18] to solve this problem.
- In practice, there are frequent cases where incorrect images are fed to the input of the algorithm. Therefore, in this paper, we propose a simple Filter Module designed to pre-filter input data before feeding the data to the main re-identification algorithm.
- We will show that due to occlusions, in practice, it will be better to use upper-body detections instead of full-body detections for person Re-ID.
- In this paper, we will introduce a fully automated data collection strategy from surveillance cameras for self-supervised pre-training to solve the lack of data to train re-identification algorithms and increase the generality of neural networks for working with real-world data in practice.

## 2. Related Work

There are three directions in the task of person Re-ID: classical person re-identification, clothing-change person re-identification, and visible–infrared person re-identification. In this paper, classical person re-identification is considered. Despite the fact that the problems differ in conditions, the ideas of the algorithms solving them may be similar. Therefore, it is useful to study methods for solving all problems. Further, some approaches to solving these problems will be considered. Furthermore, in this paper, it is proposed to use self-supervised pre-training to improve the quality of person Re-ID (Section 3.5.2); therefore, in this section, some existing approaches for self-supervised pre-training will also be considered.

### 2.1. Classical Person Re-Identification

The classical person re-identification involves searching for a person on different cameras without taking into account the possible change in clothes. This area has received a special development with the growing popularity of convolutional neural networks [10,11,19,20,21,22]. Furthermore, recently, works related to the problem under consideration have begun to appear, in which transformer-based architectures are used [23,24,25,26]. Due to the fact that there are problems with data in the task of person Re-ID (Section 1), methods based on unsupervised learning have recently begun to develop. There are two types of unsupervised re-identification algorithms: unsupervised domain adaptation and fully unsupervised. Algorithms from the first class [27,28,29] attempt to transfer knowledge from existing labeled data to unlabeled target data. Fully unsupervised methods [30,31,32] imply model training, in which there is no markup at all.

### 2.2. Clothing-Change Person Re-Identification

The idea of the clothing-change person re-identification is to find the person depicted in the query image among other images obtained from different cameras, provided that people can change clothes at different times. There are approaches that use keypoints of the human body to solve this problem [33], as well as human contour sketches [34]. Among other things, there are works [35,36] in which knowledge about the shape of the body, extracted from human silhouettes, is used for person Re-ID. Furthermore, one of the distinguishing factors of each person is gait, so there are algorithms that try to re-identify people by using video recordings [37,38].

### 2.3. Visible-Infrared Person Re-Identification

Modern surveillance systems often operate in two modes: visible mode during the day (RGB images) and infrared mode at night (grayscale images). This creates an additional challenge beyond classical re-identification. An additional task involves matching the visible image of the target with the infrared image of the corresponding person. This cross-modal image matching task is called visible–infrared person Re-ID. In [39,40,41], two separate convolutional neural networks are employed as backbones for feature extraction from infrared and visible images. Based on the extracted features, the similarity between the two images is calculated, and then optimization occurs, during which the similarity increases for pairs with the same person and decreases otherwise. There are also papers [42,43] that propose augmentations that mix RGB and grayscale images.

### 2.4. Self-Supervised Pre-Training

The main idea of the self-supervised pre-training is to learn robust representations without expensive labels or annotations. Most self-supervised approaches [44,45,46] are based on the idea of contrastive learning, where two augmentations of the same image come closer to each other in space and move away from other images. Research has shown that for better self-supervised pre-training, a large number of negative examples are needed. In [47], the authors propose using a large batch for self-supervised pre-training to increase the number of negative samples. In another work [48], all embeddings of all images from the dataset are stored in the memory bank. However, this approach is bad from the point of view of memory consumption, so in [49,50], using a momentum encoder and a queue-like memory bank to dynamically update negative samples was proposed. In [51], authors propose using variance, invariance, and covariance regularization to learn robust representations.

## 3. Proposed Approaches

**Figure 1:**
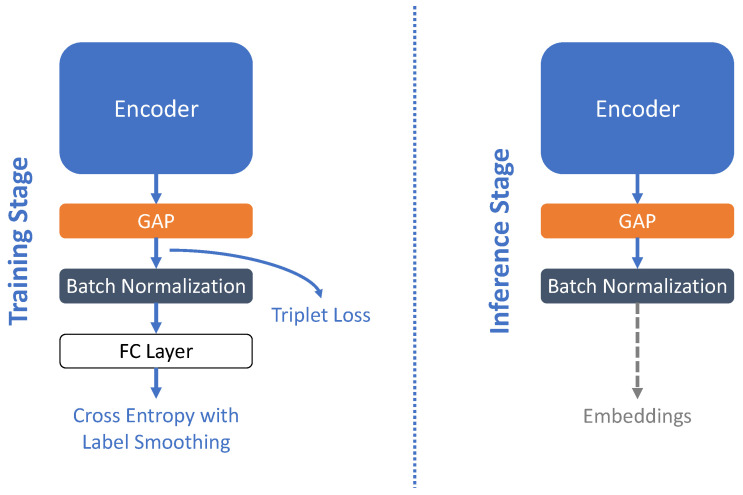
Scheme of the baseline. At the training stage, the person classification problem is solved. At the inference stage, only embeddings obtained after the batch normalization layer are used to compare images using cosine similarity.

To demonstrate the effectiveness of the approaches proposed in this paper, we use a modified version of the neural network from [10] as a baseline (Figure 1). This baseline is simple to implement and is an ideal option for subsequent improvements. Despite its simplicity, the baseline has competitive performance in benchmarks for the task of person re-identification. Furthermore, it is important to note that all the proposed approaches can be applied to more advanced re-identification algorithms.

### 3.1. Using Larger and Advanced Neural Networks as the Encoder

This paper hypothesizes that the use of larger and more advanced neural networks as the encoder can improve the quality of person Re-ID. It can only be verified experimentally (Section 4.3.1); however, the use of larger neural networks can significantly increase the computational costs and the running time of the algorithm, but this is unacceptable for practical use. Therefore, a more difficult task is finding a tradeoff between the quality of the encoder and its performance.

In this paper, we propose replacing the ResNet50 [52], used in baseline as the encoder, with Res2Net50 [53] because:

- Res2Net has an increased receptive field due to the replacement of one convolutional layer in the residual block in ResNet with a group of smaller ones that are interconnected with each other; moreover, an increased receptive field may favorably affect the quality of person Re-ID;
- Res2Net shows higher accuracy in the classification problem on ImageNet [54] than ResNet;
- The use of Res2Net as the encoder in baseline does not greatly affect the computational complexity of the algorithm. The time measurements presented in Table 1 confirm this.

Thus, Res2Net50 can improve the quality of solving the problem under consideration and at the same time slightly increase computational costs.

### 3.2. Improving the Stability of the Algorithm to Image Changes and Noise

In real-world scenarios, due to occlusions or detector mistakes, there may be cases where the detector finds only a part of the human body. The use of such detections for re-identification can seriously reduce the quality of person Re-ID. Therefore, current re-identification algorithms become unstable with image changes and noise because the slightest changes in images can seriously change the distribution of neural network outputs.

To solve this problem, in our previous work [57], we proposed the new Random Size Augmentation, which randomly changes the size of the area for re-identification during algorithm training. In this paper, we propose an improved approach to solving this problem—the use of AugMix augmentation, as well as JS Loss instead of Triplet Loss [58]. Together, the mentioned augmentation and loss function pursue the following idea: minimize the Jensen–Shannon divergence of the posterior distributions for the original image and its augmentations:

(1) $$\begin{array}{cc} M & =\frac{p_{orig}+p_{augmix_{1}}+p_{augmix_{2}}}{3}, \end{array}$$

(2) $$\begin{array}{cc} JS(p_{orig},p_{augmix_{1}},p_{augmix_{2}}) & =\frac{KL[p_{orig}||M]+KL[p_{augmix_{1}}||M]+KL[p_{augmix_{2}}\left||M\right]}{3}, \end{array}$$

where $p_{orig}$, $p_{augmix_{1}}$, and $p_{augmix_{2}}$ are the neural network output distribution for the original image and its two AugMix augmentations, respectively. KL represents the Kullback–Leibler divergence.

Thus, the distribution of neural network outputs for modified images should become similar to the distribution of neural network outputs for the original image. This makes the algorithm less sensitive to image changes and noise and improves the quality of the solution of the problem under consideration. This is also confirmed by the experimental estimate given in Section 4.3.2.

### 3.3. Filter Module

**Figure 2:**
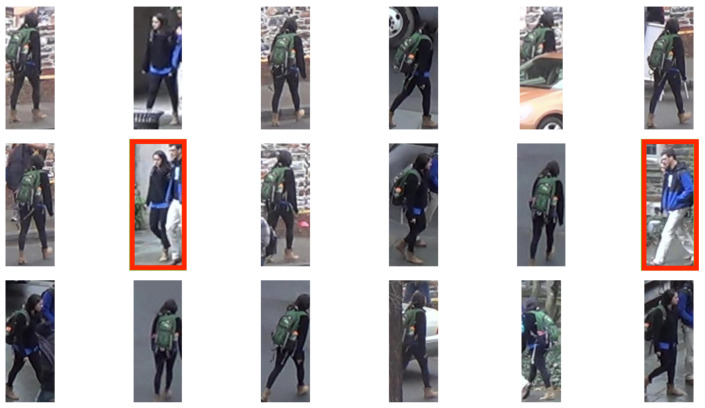
An example of a sequence of images from the track of a girl in a black sweatshirt. Incorrect images are highlighted in red, while correct images are not highlighted. *Explanations:* the eighth image is marked as incorrect since it is difficult to identify the person you are looking for. The twelfth image is highlighted as incorrect since the desired person is strongly occluded by another person. There is a high probability of an error in the re-identification algorithm on these images, so it is worth evaluating the suitability of each of the images for subsequent analysis in advance.

In practice, as noted above in Section 3.2, detection errors and errors of other components of the video analytics system are possible. Therefore, there is a possibility that the re-identification algorithm will obtain a deliberately incorrect image (for example, an image without a person) at the input. Furthermore, in practice, most often for one person there are several images at once, so it is useful to evaluate the suitability of each of the images for subsequent re-identification (Figure 2).

**Figure 3:**
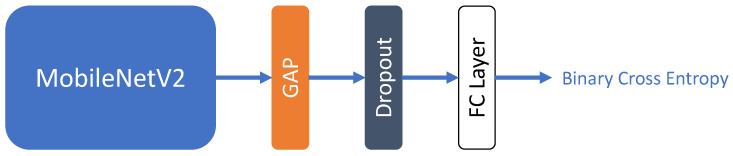
Scheme of the proposed Filter Module. This neural network has a simple architecture since it performs a secondary function and should not significantly increase computational costs of the entire pipeline in practice.

In this paper, we propose a new simple Filter Module, which is a lightweight neural network binary classifier based on MobileNetV2 [59] (Figure 3). The Filter Module predicts the suitability of an image for subsequent re-identification. That is, at the inference stage, incorrect input images are filtered before they are fed to the neural network for Re-ID.

Thus, if only correct data are fed to the input of the re-identification algorithm, then it will be able to give only confident answers, which is a key point since most of the current Re-ID algorithms do not take into account the correctness of the input images and try to make a prediction even for those examples on which this is impossible to do, and it is undesirable both in the theoretical understanding of the problem and in the practical one.

### 3.4. Upper-Body Re-ID

**Figure 4:**
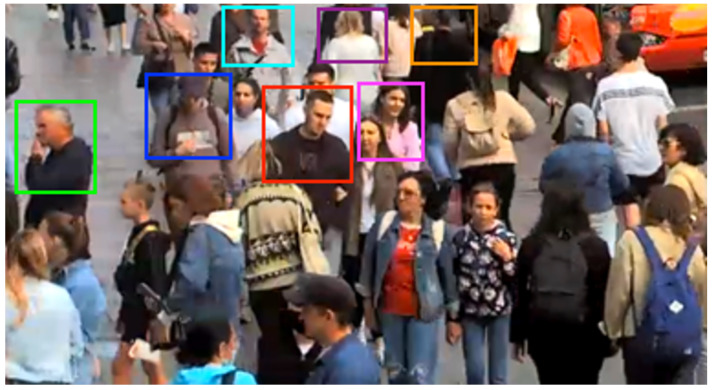
An example of a frame from a surveillance camera demonstrating the fact that upper-body detections have greater visibility than full-body detections (bounding boxes are drawn for clarity).

Re-identification methods are being implemented in video surveillance systems, where large crowds of people are dealt with most often. Due to the large crowds, most of the human body, which is used for Re-ID, is not visible (Figure 4). Using full-body person re-identification in such a scenario can lead to poor performance of the algorithm due to occlusions and detector errors. Therefore, in this paper, we propose an alternative approach, in which the input to the Re-ID algorithm is not the detection of a full body (full-body Re-ID), as is customary in classical methods, but the detection of the upper body of a person (upper-body Re-ID).

The validity of this approach was confirmed by the corresponding experiments in our previous works [57,60] on the implementation of re-identification algorithms in tracking algorithms for estimating the waiting time in queues.

### 3.5. Fully Automated Data Collection Strategy and Self-Supervised Pre-Training

As mentioned in Section 1, the lack of sufficient data to train person re-identification algorithms is one of the main problems that researchers have to face when solving this task.

This paper considers an approach to improve the quality of re-identification through self-supervised pre-training. However, for high-quality pre-training of the neural network, it is necessary to have a large and diverse dataset of images of people. Therefore, in this paper, we propose a fully automated strategy for collecting such a dataset.

#### 3.5.1. Fully Automated Data Collection Strategy

The proposed automated data collection strategy for self-supervised pre-training consists of the following steps:

1. Using the tracking algorithm [57], the tracks of the movement of people are built on video recordings collected from open sources;
2. Using the obtained tracks, images of people are cropped from video frames of video recordings;
3. After that, the crops of people are filtered automatically: false detections and cases when people are not moving during the whole video are removed, etc.

The suggested approach enables the collection of large volumes of data for neural network pre-training from open sources with minimal human involvement. By utilizing a tracking algorithm, the resulting dataset comprises multiple examples for each individual included in the dataset, which positively impacts the pre-training quality of the neural network for the person re-identification task. Furthermore, considering that each track corresponds to one person, automatic annotation can be obtained, facilitating self-supervised pre-training as well.

Section 4.1.3 provides information about which dataset for self-supervised pre-training was collected using the proposed strategy.

#### 3.5.2. Self-Supervised Pre-Training

Most often, computer vision researchers working with neural networks do the following: a neural network pre-trained on a large ImageNet dataset for the classification task is taken, and this network is fine-tuned on target data (for example, images of people) to solve the final task.

In this paper, we hypothesize that if we somehow pre-train the neural network on data that are most similar to the target (in this case, on crops of people) rather than images of 1000 classes from ImageNet, then after fine-tuning this network on data for the final task, it is possible to achieve a better quality of its solution. This paper proposes achieving this through self-supervised pre-training.

Most self-supervised approaches are based on the idea of contrastive learning. The essence of contrastive learning lies in the fact that the neural network is trained to bring together positive examples (images of the same class) in space and move negative examples (respectively, images of different classes) away from each other, which is achieved due to *Contrastive Loss:*

(3) $$Contrastive\;Loss=-\log\left(\frac{\exp\left(\frac{q\cdot k^{+}}{\tau }\right)}{\exp\left(\frac{q\cdot k^{+}}{\tau }\right)+\sum_{i=0}^{K-1}exp\left(\frac{q\cdot k_{i}^{-}}{\tau }\right)}\right),$$

where *q* is the embedding for the query image, $k^{+}$ and $k^{-}$ are the embeddings for positive and negative of the query image examples, respectively. τ is the temperature parameter; in this work, τ=0.07.

**Figure 5:**
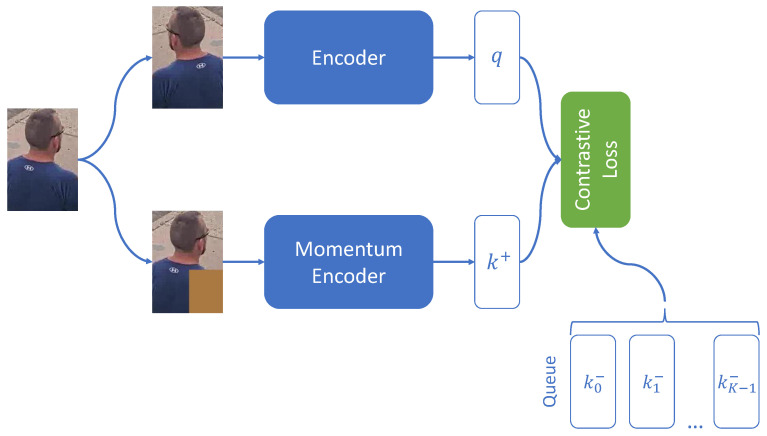
Scheme of the MoCo v2 strategy.

In this paper, the MoCo v2 strategy [50] is used as an algorithm for self-supervised pre-training. Figure 5 shows a scheme of this method. Its idea is the following:

- Two augmentations are applied to the input image;
- Then one augmented image goes to the encoder, while the other goes to the momentum encoder;
- The outputs of the encoder and momentum encoder are embeddings that are used in the calculation of contrastive loss (two augmentations are taken as positive examples, while examples from the queue are considered as negative);
- Embedding received from the momentum encoder is added to the end of the queue, and the queue is built according to the FIFO strategy;
- The momentum encoder weights are updated by momentum averaging the encoder weights.

Thus, it seems possible to pre-train the neural network to distinguish people from each other without any markup. This task is somewhat simpler than person re-identification. However, it can be assumed that for the problem under consideration, it is more efficient to fine-tune a network that can somehow distinguish people, rather than classify various objects represented in ImageNet. This hypothesis is supported by the experimental evaluation presented in Section 4.3.4.

## 4. Experiments

### 4.1. Datasets

In the experiments conducted in this paper, three types of datasets were used: datasets designed for training and testing the algorithm for the person re-identification task, data for training and testing the Filter Module, as well as data for self-supervised pre-training. Next, all the datasets used in this work will be described.

#### 4.1.1. Datasets for Person Re-ID

In this paper, the well-known datasets Market-1501 [1], DukeMTMC-reID [2], and MSMT17 [3] were used to train and test the proposed person Re-ID algorithms. All these datasets consist of full-body images of people taken from multiple cameras. For experiments related to upper-body re-identification, in this paper, we used modified versions of the datasets that were proposed in our previous work [57].

#### 4.1.2. Datasets for Filter Module

Since the idea of filtering input data during the testing of the re-identification algorithm was not previously mentioned in other works, we had to face the problem associated with the lack of datasets for training and testing the Filter Module. For this reason, we decided to develop criteria for classifying images into correct and incorrect.

The image can be considered as correct if it meets the following conditions:

- There should be a person in the image;
- It should be obvious from the image which person it belongs to;
- The desired person should not be overlapped too much by other people or objects;
- The image should not be heavily cropped and the person in the image should not be too close;
- The desired person and the colors of the clothes should be distinguishable in the image.

**Figure 6:**
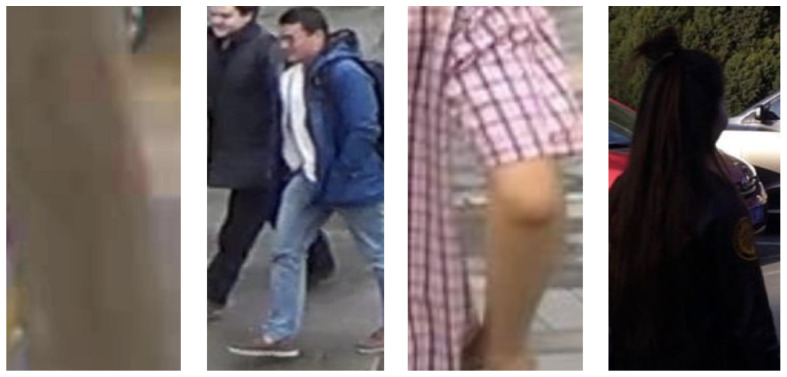
Examples of incorrect images on which there is a high probability of an error of the re-identification algorithm. In the first image, there is no person; in the second, there are two people at once (i.e., it is not clear which of them needs to be re-identified); in the third, only a part of the body, which is very difficult to re-identify, got into the image; in the fourth, the image is too dark, so it is almost impossible to distinguish the person and the colours of clothes.

According to the above criteria, the dataset MSMT17 for person Re-ID was manually marked up. Figure 6 shows examples of incorrect images.

#### 4.1.3. Datasets for Self-Supervised Pre-Training

**Figure 7:**
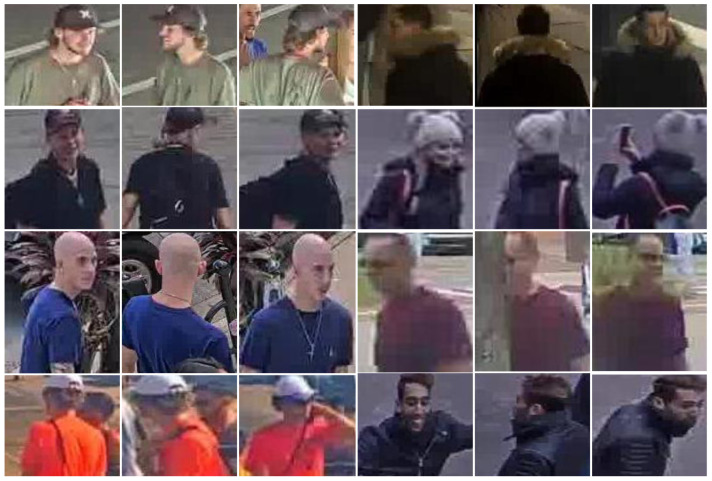
Examples of images in the collected dataset for self-supervised pre-training.

As part of this work, 371 videos from surveillance cameras publicly broadcast on the Internet were selected, with a total duration of more than 2500 h. For 2.5 weeks, all videos were processed according to the fully automated data collection strategy described in Section 3.5.1, and as a result, about 11.5 million crops of about 980 thousand people were received (Figure 7).

Table 2 shows a comparative assessment of the obtained dataset for self-supervised pre-training with well-known datasets for the person Re-ID task.

### 4.2. Metrics

In the re-identification task, two metrics are most often used to determine the quality of algorithms: $Rank_{N}$ and mAP. The first one is calculated as follows:

(4) $$\begin{array}{cc} Rank_{N} & =\frac{\sum_{q\in Q}Acc_{N}\left(q\right)}{\left|Q\right|}, \end{array}$$

where *Q* is the set of all query images involved in testing, and $Acc_{N}$ takes the following values:

(5) $$\begin{array}{cc} Acc_{N}\left(q\right) & =\left\{\begin{array}{cc} 1, & \begin{array}{c} \mathrm{if}\;\mathrm{the}\;\mathrm{Top}\text{-}N\;\mathrm{of}\;\mathrm{the}\;\mathrm{gallery}\;\mathrm{images}\;\mathrm{issued}\;\mathrm{by}\;\mathrm{the}\;\mathrm{algorithm}\\ \mathrm{includes}\;\mathrm{an}\;\mathrm{image}\;\mathrm{with}\;\mathrm{the}\;\mathrm{same}\;\mathrm{ID}\;\mathrm{as}\;\mathrm{the}\;\mathrm{image}\;q \end{array}\\ 0, & \mathrm{else} \end{array}\right. \end{array}$$

In the $Rank_{N}$ metric, the parameter *N* means the number of gallery images with the highest confidence of the Re-ID algorithm for the query image, among which there should be an image with the desired person ID.

As for the mAP metric, it is calculated as follows:

(6) $$\begin{array}{cc} mAP & =\frac{\sum_{q\in Q}AP\left(q\right)}{\left|Q\right|}, \end{array}$$

where AP(q) is the area under the Precision–Recall curve for the image *q*. *Precision* and *Recall*, in turn, are defined as:

(7) $$\begin{array}{cc} Precision & =\frac{TP}{TP+FP}, \end{array}$$

(8) $$\begin{array}{cc} Recall & =\frac{TP}{TP+FN}, \end{array}$$

where TP is True Positive matches, FP is False Positive matches, and FN is False Negative matches.

### 4.3. Experimental Results

There are two types of procedures for testing person re-identification algorithms: standard test protocol and cross-domain Re-ID. In the first case, we train and test the algorithm on the same dataset. In cross-domain Re-ID, we train the algorithm on one dataset and test it on another (the domain gap problem mentioned in Section 1).

Cross-domain Re-ID is the closest to the actual use of the algorithm in practice because, in practice, it is often impossible to train a neural network on the data on which it will be applied. Therefore, in this paper, during the experimental evaluation, the main emphasis was placed on tests in a scenario where the algorithm is trained on one dataset and tested on another.

#### 4.3.1. Using Larger and Advanced Neural Networks as the Encoder

As can be seen from the experimental evaluation presented in Table 3, the hypothesis that larger and more advanced neural networks are better able to solve the re-identification problem, as put forward in Section 3.1, has been confirmed.

Table 4 presents an experimental evaluation showing the impact of using larger and more advanced neural network architectures on the quality of cross-domain Re-ID. As can be seen from the experimental results, the use of such neural networks is justified, especially in the case of cross-domain re-identification. Moreover, if we take into account the time measurements presented in Table 1, the use of Res2Net50 as the encoder in practice will not particularly affect the performance of the algorithm as a whole, but it will improve its quality in the Re-ID task.

Furthermore, from the results presented in Table 4, we can conclude that the use of larger datasets for training can significantly improve the results of person re-identification in the scenario when the algorithm is trained on one dataset and tested on another. This is very useful in practice, so in all the following experiments in this paper, training will be carried out with the large dataset MSMT17 Merged, and testing will be conducted with other smaller datasets.

#### 4.3.2. Improving the Stability of the Algorithm to Image Changes and Noise

The experimental evaluation presented in Table 5 confirms the idea that the use of AugMix augmentation and JS loss reduces the sensitivity of the algorithm to image changes and noise and also improves the overall quality of solving the re-identification problem.

This result is important since most of the existing Re-ID algorithms are unstable with image changes, which often occur in practice due to detector errors, occlusions, and many other factors.

#### 4.3.3. Filter Module

As mentioned earlier in Section 3.3, bad images can be fed to the input of the Re-ID algorithm, due to which the overall quality of re-identification may deteriorate. In this work, in the inference stage for preliminary filtering of such images, the Filter Module was proposed. In this series of experiments, the influence of the Filter Module, depending on the thresholds of the values of image suitability issued by it, on the quality of person Re-ID is considered.

As can be seen from the results of all experiments (Table 6 and Table 7), pre-filtering of the gallery and the set of query images has a positive effect on the metrics used in re-identification. Moreover, in most cases, we are talking about an increase in quality by several percent for both metrics, with a very small decrease in the volume of test sets. This suggests that the Filter Module does not just reject random images, but really looks for images unsuitable for person Re-ID and does not feed them to the input of the main Re-ID algorithm.

Thus, the Filter Module proposed in this paper can be used in practice to predict the suitability of images for re-identification and improve the quality of person Re-ID without a significant increase in computational costs due to the lightweight architecture of the Filter Module.

#### 4.3.4. Upper-Body Re-ID and Self-Supervised Pre-Training

In this paper, in addition to the classical full-body re-identification, the algorithm based on upper-body detections is proposed (Section 3.4). The experimental results presented in Table 8 show that the use of Res2Net50 as the encoder, as well as the use of AugMix augmentation and JS Loss during algorithm training, can significantly improve the quality of upper-body Re-ID (as well as for full-body re-identification).

Furthermore, the conducted experiments show that self-supervised pre-training on the data collected using the proposed fully automated data collection strategy (Section 3.5 and Section 4.1.3) helps to improve the quality of upper-body Re-ID. Thus, the hypothesis put forward earlier in Section 3.5.2 was experimentally confirmed that if the neural network is pre-trained on data that are most similar to the target rather than images from ImageNet, then after fine-tuning this network on data for the final task, it becomes possible to achieve a better quality solution.

Moreover, during the experiments, two no less important and interesting observations were obtained for the problem of person re-identification:

- Self-supervised pre-training on images of people allows us to speed up the neural network training procedure for re-identification by 1.5 times. That is, a result identical to the fine-tuning of the network pre-trained in supervised mode on ImageNet can be achieved in fewer epochs with the fine-tuning of the network pre-trained in self-supervised mode.
- From the dataset described in Section 4.1.3, a subsample was randomly selected (about 300 thousand images of people). Further, the neural network was pre-trained on it in self-supervised mode. After that, it was found that fine-tuning the neural network pre-trained in self-supervised mode on a relatively small dataset can be more efficient in terms of re-identification quality than fine-tuning the network pre-trained in supervised mode on the whole ImageNet.

Thus, the upper-body Re-ID algorithm proposed in this paper, despite using a smaller area of the human body for analysis, has competitive results and is the preferred option in scenarios when it comes to video analytics on scenes with a large crowd of people.

## 5. Discussion

### 5.1. Comparison with Other Re-ID Methods

The main goal of our work is not to get ahead of the state-of-the-art re-identification algorithms but to study the main difficulties of the problem under consideration that arise in practice. As mentioned earlier, this paper proposes approaches (Section 3) that can be integrated with almost any of the popular modern person Re-ID algorithms in order to improve their quality while maintaining their computational complexity. On the example of a simple baseline [10], the consistency of all proposed approaches was demonstrated (Section 4.3).

However, despite the simplicity of the chosen baseline, with the help of the proposed approaches, it was possible to make it even more competitive in a cross-domain scenario with other more complex and advanced re-identification algorithms (Table 9). At the same time, the computational complexity of a simple baseline remained practically unchanged at the inference stage.

As can be judged from the experimental results presented in Table 9, the proposed approaches can significantly improve the quality of current algorithms while maintaining their computational complexity. Thus, using the methods proposed in this paper, it is possible to improve the quality of almost any of the popular modern algorithms for person re-identification. Demonstrating such an improvement is one of our future research goals (Section 5.4).

### 5.2. Practical Use of the Proposed Methods

Re-identification algorithms can be used in practice for their primary purpose—to search for people by query image in the database—and as components of other algorithms. For example, in [57,60,62], Re-ID algorithms are used as an integral part of the tracking algorithm for matching detections.

In our previous works [57,60], we also used re-identification methods to create a tracking algorithm capable of working with low detection frequency in order to solve the practically important task of estimating the waiting time in queues. That is, Re-ID methods can reduce the frequency of detection and thereby significantly reduce computational costs for the entire pipeline.

Returning to the primary purpose of re-identification algorithms, in most cases, the tracks of the movement of people are built on each surveillance camera. In most cases, this is completed in order to avoid storing a huge number of embeddings for each detection in the database, instead only a single embedding for each of the tracks, thereby facilitating the search. This approach leads to the question of how best to form this single embedding. The easiest way is to average the embeddings for all detections included in the track. However, poor detections (for example, due to detector errors or occlusions) can lead to poor performance of the Re-ID algorithm, and therefore averaging can work poorly. However, using the proposed Filter Module (Section 3.3) allows solving this dilemma in at least two ways:

- When calculating the average embedding, it is possible to exclude those detections that have a low suitability value issued by the Filter Module;
- Among all the detections in the track, choose the best shot. The best shot can be the detection that has the highest suitability value issued by the Filter Module. Furthermore, as a single embedding for the track, take the embedding corresponding to this detection.

### 5.3. Why Is Upper-Body Re-ID Useful?

If we compare the results presented in Table 5 and Table 8, it may seem that it is always worth using the full-body re-identification algorithm and that there is no sense in the upper-body Re-ID. However, in this paper, we propose the upper-body Re-ID algorithm as a result of our research aimed at practice.

In real scenes from surveillance cameras, occlusions are very frequent, because of which, in most cases, the full body is not visible, with the exception of only its upper part (Figure 4). In the case of occlusions, both detector errors are possible (for example, several people combine into one detection) and errors of the re-identification itself. Since well-known datasets for person Re-ID do not fully cover such cases (occlusions are much less common in them), it may seem that the full-body re-identification always works better.

Thus, it is recommended to combine these algorithms. If people are clearly visible in full height, use the full-body Re-ID algorithm; otherwise, the upper-body re-identification is recommended.

### 5.4. Future Research

There are many directions for continuing research on this topic. For example, it is necessary to integrate the proposed approaches into other existing Re-ID algorithms. Furthermore, the fully automated data collection strategy proposed in this paper implies obtaining some automatic markup through the use of tracking (Section 3.5.1). In this paper, this markup was not used during self-supervised pre-training. In theory, creating a self-supervised pre-training algorithm that will use this markup can improve the quality of the final algorithm. In conclusion, as noted in Section 5.3, well-known datasets for the problem under consideration do not reflect all the problems that may arise in practice, so there is a need to collect new datasets for a more honest comparison of the full-body re-identification and the upper-body Re-ID algorithms.

## 6. Conclusions

In this paper, the main problems that arise in the practical solution of the task of person re-identification were considered. We have proposed several approaches that allow us to improve the quality of existing Re-ID algorithms, as well as preserve its computational complexity. In summary, the contributions of this paper are concluded as follows:

- We have shown that larger and advanced neural networks are able to solve the problem of person re-identification better. Furthermore, also in this paper, the encoder architecture was chosen, which improves the balance between the quality of the algorithm and its speed.
- Approaches have been proposed that make it possible to increase the algorithm’s stability in response to image changes and noise.
- We have proposed the new Filter Module designed to pre-filter input data before feeding the data to the main re-identification algorithm.
- We have shown that due to occlusions, in practice, it is better to use upper-body detections instead of full-body detections for person Re-ID.
- The fully automated data collection strategy from surveillance cameras for self-supervised pre-training has been proposed in order to increase the generality of neural networks on real-world data.

The experimental evaluation presented in this paper has shown the consistency of all the proposed approaches. Among other things, in this paper, the practical application of the proposed algorithms was considered. We believe that the results of this work can become an object for further research since they can be transferred to other computer vision tasks with a high degree of probability.

## Figures and Tables

**Table 1:** The time measurements for ResNet50 and Res2Net50 on the Intel Core i5-10600K.

Encoder	Single Core Speed *
ResNet50	202 ms
Res2Net50	224 ms

* To calculate the speed of the neural network on single core, we convert our model to ONNX [55] and run it 1000 times on one processor core using ONNX Runtime [56]. After that, we find the average speed of the neural network.

**Table 2:** Comparison of the collected dataset for self-supervised pre-training with datasets for person re-identifcation.

Dataset	Images	Persons	Location	Seasons	Camera	Resolution
Market-1501	32,668	1501	University	Fixed	Fixed	Fixed
DukeMTMC-reID	36,411	1812	University	Fixed	Fixed	Fixed
MSMT17	126,441	4101	University	Fixed	Fixed	Fixed
Our	>11.5 M	>980 K	Different	Different	Different	Different

**Table 3:** Experimental evaluation of the algorithm with different backbones (standard test protocol).

Algorithm	Market-1501		DukeMTMC-reID	
	${\mathrm{Rank}}_{1}\left(↑\right),\%$	mAP(↑),%	${\mathrm{Rank}}_{1}\left(↑\right),\%$	mAP(↑),%
Baseline (ResNet50)	94.50	85.90	86.40	76.40
With Res2Net50	**95.01**	**87.19**	**88.15**	**77.61**

The best results by metrics are highlighted in bold.

**Table 4:** Experimental evaluation of the algorithm with different backbones (cross-domain Re-ID). *Symbols:* D—DukeMTMC-reID; M—Market-1501; MSMT17 Merged—MSMT17, which combines training and test parts.

**Algorithm**	**D → M**		**MSMT17 → M**	
	${\mathrm{Rank}}_{1}\left(↑\right),\%$	mAP(↑),%	${\mathrm{Rank}}_{1}\left(↑\right),\%$	mAP(↑),%
Baseline (ResNet50)	**54.30**	25.50	58.82	30.25
With Res2Net50	54.16	**25.95**	**62.02**	**32.70**
**Algorithm**	**M → D**		**MSMT17 → D**	
	${\mathrm{Rank}}_{1}\left(↑\right),\%$	mAP(↑),%	${\mathrm{Rank}}_{1}\left(↑\right),\%$	mAP(↑),%
Baseline (ResNet50)	41.40	25.70	58.53	38.25
With Res2Net50	**44.84**	**26.67**	**63.60**	**42.59**
**Algorithm**	**MSMT17 Merged → M**		**MSMT17 Merged → D**	
	${\mathrm{Rank}}_{1}\left(↑\right),\%$	mAP(↑),%	${\mathrm{Rank}}_{1}\left(↑\right),\%$	mAP(↑),%
Baseline (ResNet50)	65.65	37.71	66.16	47.74
With Res2Net50	**69.74**	**41.32**	**70.65**	**52.37**

The best results by metrics are highlighted in bold.

**Table 5:** Experimental evaluation of the algorithm with AugMix augmentation and JS Loss (cross-domain Re-ID). *Symbols:* M—Market-1501; D—DukeMTMC-reID; MSMT17 Merged—MSMT17, which combines training and test parts.

Algorithm	MSMT17 Merged → M		MSMT17 Merged → D	
	${\mathrm{Rank}}_{1}\left(↑\right),\%$	mAP(↑),%	${\mathrm{Rank}}_{1}\left(↑\right),\%$	mAP(↑),%
Baseline	65.65	37.71	66.16	47.74
+Res2Net50	69.74	41.32	70.65	52.37
+AugMix and JS Loss	**71.08**	**43.39**	**72.51**	**53.71**

The best results by metrics are highlighted in bold.

**Table 6:** Experimental evaluation of the dependence of the quality of re-identification on the probability threshold applied to the Filter Module: the case when the neural network for Re-ID is trained on MSMT17 Merged and tested on Market-1501 (cross-domain Re-ID).

Threshold	${\mathrm{Rank}}_{1}\left(↑\right)$, %	mAP(↑), %	Gallery Reduction, %	Query Reduction, %
0.0	71.08	43.39	0.00	0.00
0.1	71.08	43.39	0.01	0.00
0.2	71.13	43.43	0.25	0.06
0.3	71.15	43.46	0.80	0.09
0.4	71.19	43.51	1.47	0.15
0.5	71.23	43.62	2.29	0.50
0.6	71.39	43.84	3.50	1.34
0.7	71.55	44.13	5.40	2.38
0.8	72.17	44.87	8.20	4.81
0.9	73.69	46.81	14.04	11.28

**Table 7:** Experimental evaluation of the dependence of the quality of re-identification on the probability threshold applied to the Filter Module: the case when the neural network for Re-ID is trained on MSMT17 Merged and tested on DukeMTMC-reID (cross-domain Re-ID).

Threshold	${\mathrm{Rank}}_{1}\left(↑\right)$, %	mAP(↑), %	Gallery Reduction, %	Query Reduction, %
0.0	72.51	53.71	0.00	0.00
0.1	72.54	53.73	0.01	0.04
0.2	72.77	54.06	0.25	0.49
0.3	73.13	54.64	0.80	1.12
0.4	73.40	55.11	1.47	1.75
0.5	73.82	55.64	2.29	2.51
0.6	74.12	56.20	3.50	3.23
0.7	74.80	57.07	5.40	4.44
0.8	75.68	58.40	8.20	6.91
0.9	77.55	60.99	14.04	12.30

**Table 8:** Experiments with upper-body Re-ID and self-supervised pre-training (cross-domain Re-ID). *Symbols:* MSMT17 Merged—MSMT17, which combines training and test parts.

Algorithm	MSMT17 Merged → DukeMTMC-reID	
	${\mathrm{Rank}}_{1}\left(↑\right),\%$	mAP(↑),%
Baseline (upper-body)	50.97	33.57
+Res2Net50	53.34	34.73
+AugMix and JS Loss	57.12	37.69
+Self-supervised Pre-training	**58.24**	**38.70**

The best results by metrics are highlighted in bold.

**Table 9:** Experimental comparison of full-body re-identification algorithms (cross-domain Re-ID). *Symbols:* M—Market-1501; D—DukeMTMC-reID; MSMT17 Merged—MSMT17, which combines training and test parts.

Algorithm	MSMT17 Merged → M		MSMT17 Merged → D	
	${\mathrm{Rank}}_{1}\left(↑\right),\%$	mAP(↑),%	${\mathrm{Rank}}_{1}\left(↑\right),\%$	mAP(↑),%
Baseline [10]	65.65	37.71	66.16	47.74
OSNet-IBN [19]	66.47	37.22	67.43	45.59
OSNet-AIN [61]	70.13	43.28	71.06	52.72
QAConv [21]	72.64	43.11	—	—
Our	71.08	43.39	72.51	53.71
Our + Filter Module	**73.69**	**46.81**	**77.55**	**60.99**

The best results by metrics are highlighted in bold.

## Data Availability

Not applicable.

## Abbreviations

The following abbreviation is used in this manuscript:

Re-ID	Re-identification
